# Extent of Visceral Pleural Invasion Affects Prognosis of Resected Non-small Cell Lung Cancer: A meta-analysis

**DOI:** 10.1038/s41598-017-01845-7

**Published:** 2017-05-08

**Authors:** Ting Wang, Chengya Zhou, Qinghua Zhou

**Affiliations:** 1 Lung Cancer Center, West China Hospital, Sichuan University, Chengdu, Sichuan China; 2 Department of Medical Oncology, Sichuan Cancer Hospital & Institute, Sichuan Cancer Center, School of Medicine, University of Electronic Science and Technology of China, Chengdu, China

## Abstract

Visceral pleural invasion (VPI) has been known to be an adverse prognostic factor in non-small cell lung cancer (NSCLC). However, the prognostic significance of extent of VPI (PL0, PL1 and PL2) remains controversial. We conduct a meta-analysis to summarize available evidence on this topic. PubMed, EMBASE, OVID and The Cochrane Library were searched for published studies from inception to May 9, 2016. A total of 16 studies were included in meta-analysis. Our results showed that patients with PL1 or PL2 had poorer overall survival compared with PL0 (HR = 1.555, 95% CI 1.399, 1.730; HR = 2.447, 95% CI 1.913, 3.130) and patients with PL2 had even poorer overall survival than PL1 (HR = 1.287, 95% CI 1.114, 1.487). Patients with PL1 or PL2 had lower 5-year survival rate than PL0 patients (OR = 0.515, 95% CI 0.415, 0.640; OR = 0.441, 95% CI 0.336, 0.579) and patients with PL2 had even lower 5-year survival rate than PL1 (OR = 0.706, 95% CI 0.545, 0.915). In conclusion, extent of VPI impacts the prognosis of resected NSCLC and VPI should be categorized as PL1 and PL2 in the terms of clinical practice and trials.

## Introduction

Lung cancer is the leading cause of cancer death worldwide. Visceral pleural invasion (VPI), since 1970s, has been adopted as a T descriptor in the TNM classification and known to be an adverse prognostic factor in non-small cell lung cancer (NSCLC)^1–3^. The 7th edition TNM staging system of lung cancer recommended the classification of pleural invasion as PL0 if the tumor does not invade past the elastic layer, as PL1 if it invades past the elastic layer, PL2 if it invades to the pleural surface and PL3 if it invades to the parietal pleura^4^. PL1 and PL2 were defined as VPI and PL0 was defined as without VPI.

However, the International Association for the Study of Lung Cancer (IASLC) team didn’t analysis and validate the prognosis of PL status in the 7th TMN classification of lung cancer because of insufficient data to be submitted^3^. Most studies investigated the prognostic value of VPI without distinguishing the extent of VPI (PL1 and PL2)^5–10^. It is still unclear whether PL1 and PL2 are equivalent and whether they should be combined to define VPI, or how tumors with PL1 and PL2 should be classified. Recently, Chan YL and associates reported resected NSCLC patients with PL2 had significant worse survival than those with PL1 and suggested PL2 to be a potential indication for adjuvant chemotherapy^11^. Likewise, Hung J. J. *et al*. reported patients with PL2 had significantly worse overall survival and lower probability of freedom from recurrence than those with PL1 after resection of node-negative NSCLC^12^. And some other studies also reached positive results^13–15^. Contrary to the studies mentioned above, there were some other studies that didn’t find the survival difference between PL1 and PL2 patients^16–24^. Thus, the evidence on this topic remains controversial.

Our previously study has demonstrated that VPI is a consistent adverse prognostic factor in stage I NSCLC patients^25^. In this study, we focused on the prognostic significance of PL0, PL1 and PL2 and aimed to answer the question whether PL2 has worse prognosis than PL1 in resected NSCLC patients.

## Methods

### Eligibility criteria

Two investigators (Ting Wang and Chengya Zhou) independently evaluated the potential articles through reading titles, abstracts and full text to decide eligibility of studies. The studies were considered to be included if:(1) original cohort studies published from inception to May 9, 2016 without language restrictions; (2) studies comparing survival outcomes between resected NSCLC patients with PL0, PL1 or PL2; (3) studies reporting at least one survival outcome such as overall survival (OS), 5-year survival rate or recurrence free survival (RFS); and (4) study participants having been pathologically diagnosed NSCLC after resection. The following studies were excluded if: (1) studies including cancers other than NSCLC; (2) studies containing no available survival data for analysis; (3) participants in studies receiving neoadjuvant therapy; and (4) studies published as review, letter or other non-original types.

### Search strategy

An electronic search in PubMed, EMBASE, OVID and the Cochrane Library were conducted from inception to May 9, 2016. The following key words in combination as medical subject heading terms and text words were used: “lung cancer” and “visceral pleural invasion”. Potentially relevant articles were identified by reading titles and abstracts. The full texts of the relevant articles were read to determine whether they met the inclusion criteria. The references were also searched to identify relevant studies.

### Quality assessment

For cohort studies, the 9-star Newcastle-Ottawa Quality Assessment Scale was used to assess the risk of bias^26^. This scale is an 8-item instrument that allows for assessment of patient population and selection, study comparability, follow-up, and outcome. Interpretation of the scale is performed by awarding points for high-quality elements. Studies with 5 or more stars were defined as high-quality studies and were included.

### Statistical analyses

Data was extracted using a unified form and study information including author name, study year, study area, sample size, tumor size, pathologic type, staining method, adjuvant therapy, 5-year survival rate and hazard ratio (HR) of OS or RFS were collected. If the HR was not reported in the original article, we would calculate HR from reported data or survival curves according to the methods described by Tierney *et al*.^27^. For studies reported 2, 3, or 4-year survival instead of 5-year survival, the 5-year survival rate would be calculated, if possible, according to the survival curves too. Statistical heterogeneity among studies was examined using the Cochrane Q test by calculating the I^2^ value^28^. The I^2^ value greater than 50% or p value less than 0.05 were considered to represent significant heterogeneity. The pooled HR and the 95% confidence interval (CI) were calculated using the Z test. The pooled HR and the 95% CI were calculated using the Mantel-Haenszel formula (fixed-effect model) when heterogeneity was not detected (p > 0.05), or using the DerSimonian-Laird formula (random-effect model) when heterogeneity was significant (p < 0.05)^29^. Subgroup analyses were conducted by confounding factors to detect the source of heterogeneity and assess the effect of those factors on results. Publication bias was evaluated using the funnel plot and the Begg’s test^30^. Influence analyses were conducted to access how robust the pooled estimators were by removing individual studies. An individual study was suspected of excessive influence if the point estimate of its omitted analysis was outside the 95% CI of the combined analysis. Statistical analyses were performed with Comprehensive Meta Analysis professional version 2.2 (Biostat Inc, Englewood NJ, www.meta-analysis.com).

## Results

### Study selection

**Figure 1:**
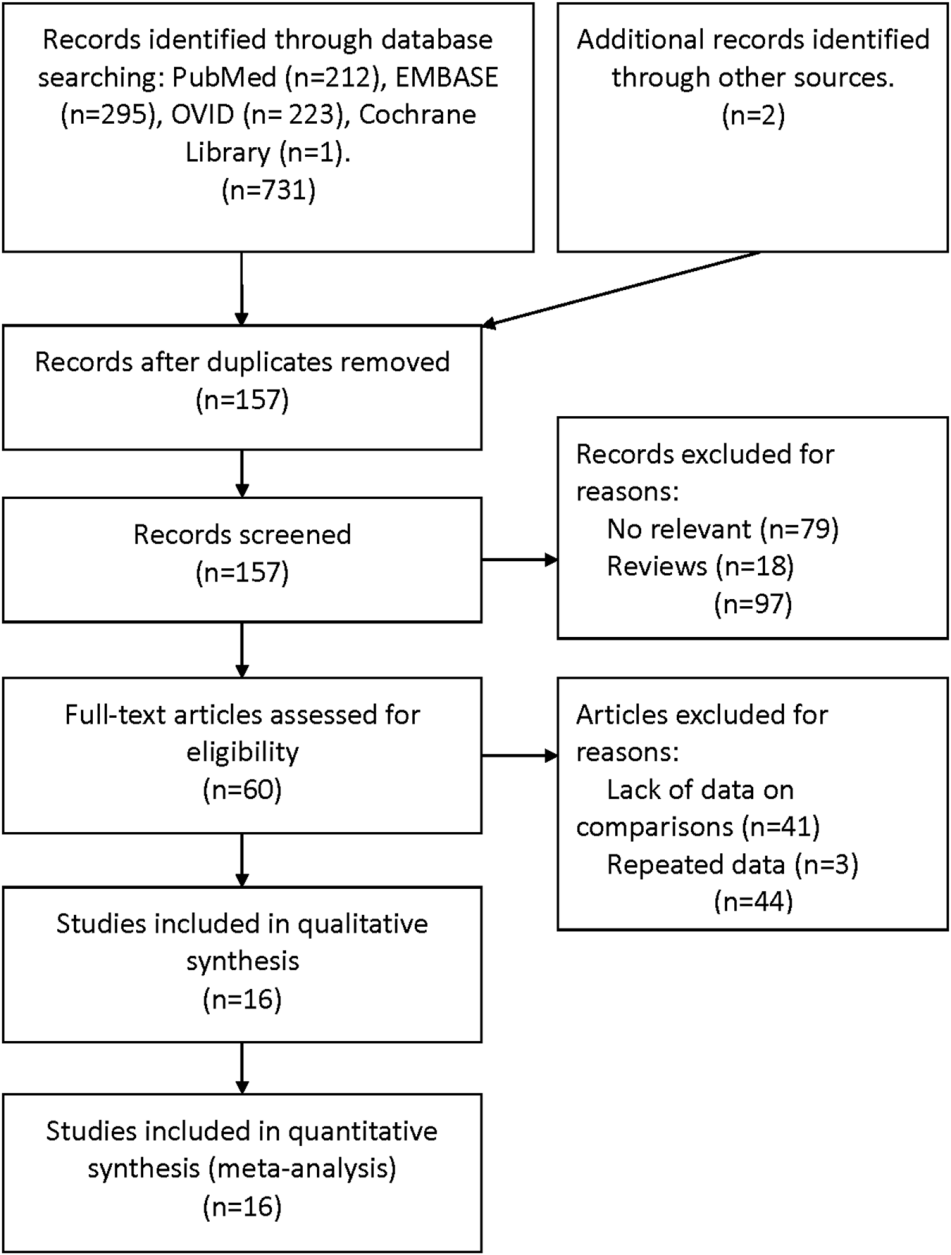
Flowchart of the identification of relevant studies.

Electronic search identified 731 potentially relevant references. Additional 2 references were further identified by checking the reference list. 576 duplicates or clearly irrelevant references were excluded through reading the abstracts. 157 references were read in full and 97 references were excluded for irrelevance, 41 references were excluded for lack of data on comparisons or outcomes and 3 references were excluded for repeated data. Finally, 16 references fulfilled the inclusion criteria and provided data for the meta-analysis^11–24, 31, 32^ (Fig. 1).

### Characteristics of included studies

All 16 included articles were cohort studies published from 2004 to 2015^11–24, 31, 32^. This study included 16916 patients, 3667 (21.7%) of them had PL1 and 1512 (8.9%) had PL2. Potential confounders, such as tumor size, age, gender, history of smoking, tumor differentiation, and type of operation were reported and adjusted in most of these studies. The quality score of included studies ranged from 6 to 8 stars. Hazard ratios of overall survival were available in 15 included studies, 5-year survival rates were reported in 14 included studies and hazard ratios of recurrence-free survival were available in three included studies. Characteristics of the included studies are listed in Table 1.

**Table 1:** Characteristics of included studies.

Study	Period	Area	Median Age (year)	Tumor Stage	Median Follow-up (year)	Patient Number			VPI Rate (%)	Staining Method	Pathologic Type	Type of Resection	Adjuvant Therapy	Quality Score
						PL0	PL1	PL2						
Osaki T.^20^	1992–2001	Japan	66.5	I-III	2.9	345	110	19	27.2%	H&E and elastic staining	AC, SCC, LCC, ASC and others	Pneumonectomy, bilobectomy, lobectomy and segmentectomy/wedge	NA	7
Shimizu K.^23^	1979–2001	Japan	65	I-III	NA	1055	271	81	25.0%	H&E and elastic staining	AC, SCC, LCC and ASC	Pneumonectomy lobectomy and segmentectomy	NA	7
Sakakura N.^15^	1982–2000	Japan	62	I-III	NA	427	462	199	60.8%	H&E	AC, SCC, LCC and ASC	Lobectomy pneumonectomy partial resection and segmentectomy	NA	7
Hsu C. P.^17^	1997–2006	Taiwan	67	I	8.6	96	42	134	64.7%	H&E	AC, SCC and Others	Pneumonectomy bilobectomy and lobectomy segmentectomy wedge resection	No	8
Shim H. S.^22^	1990–2005	Korea	61	I-III	NA	680	86	141	25.0%	H&E and elastic staining	AC, SCC, LCC and ASC	Pneumonectomy bilobectomy, lobectomy and segmentectomy	NA	7
Kawase A.^18^	1979–2006	Japan	66	I-III	NA	1693	417	150	25.1%	H&E and elastic staining	AC, SCC, LCC, ASC and others	Pneumonectomy lobectomy segmentectomy	NA	7
Yilmaz A.^24^	2000–2009	Turkey	NA	I-IV	NA	96	34	37	42.5%	H&E and elastic staining	AC and SCC	Lobectomy/bilobectomy pneumonectomy lobectomy + chest wall resection and pneumonectomy + chest wall resection	No	7
Chang Y. L.^11^	1990–2008	Taiwan	64	I-III	NA	NA	151	170	—	H&E and elastic staining	AC, SCC and ASC	Lobectomy	Yes	7
Hung J. J.^12^	1990–2006	Taiwan	67	I-II	4.5	NA	300	55	—	H&E and elastic staining	AC, SCC, LCCs and others	Pneumonectomy bilobectomy, lobectomy and sublobar resection	No	7
Kudo Y.^14^	2000–2007	Japan	66	I-III	4.6	692	132	62	21.9%	H&E and elastic staining	AC, SCC, LCC and others	Pneumonectomy bilobectomy and lobectomy	Yes	7
Hung J. J.^31^	2001–2008	Taiwan	65.2	I	4.5	115	122	29	56.8%	H&E	AC	Bilobectomy lobectomy and sublobar resection	Yes	6
Kawase A.^13^	2004	Japan	67	I-II	>5	3606	727	219	20.8%	H&E	AC, SCC, LCC, ASC and others	Pneumonectomy bilobectomy, lobectomy	Yes	8
Nitadori J.^19^	2000–2008	USA	68	I	3.6	685	81	11	11.8%	H&E and elastic staining	AC	Bilobectomy lobectomy, segmentectomy and wedge resection	No	7
Oyama M.^21^	1997–2004	Japan	65	I-III	5.5	1006	261	86	25.6%	H&E and elastic staining	AC, SCC and others	Pneumonectomy and lobectomy	NA	7
Kachala S. S.^32^	1995–2009	USA	67.9	I-III	3.1	779	336	77	34.6%	H&E	AC	NA	Yes	7
Adachi H.^16^	2005–2007	Japan	67.2	I-III	5.4	462	135	42	27.7%	H&E and elastic staining	AC, SCC, LCC and others	Pneumonectomy bilobectomy, lobectomy	Yes	8

*VPI: visceral pleural invasion; H&E: hematoxylin-eosin staining; AC: adenocarcinoma; SCC: squamous cell carcinoma; LCC: large cell carcinoma; ASC: adenosquamous carcinoma; NA: not available.

### Impact of extent of VPI on overall survival

**Figure 2:**
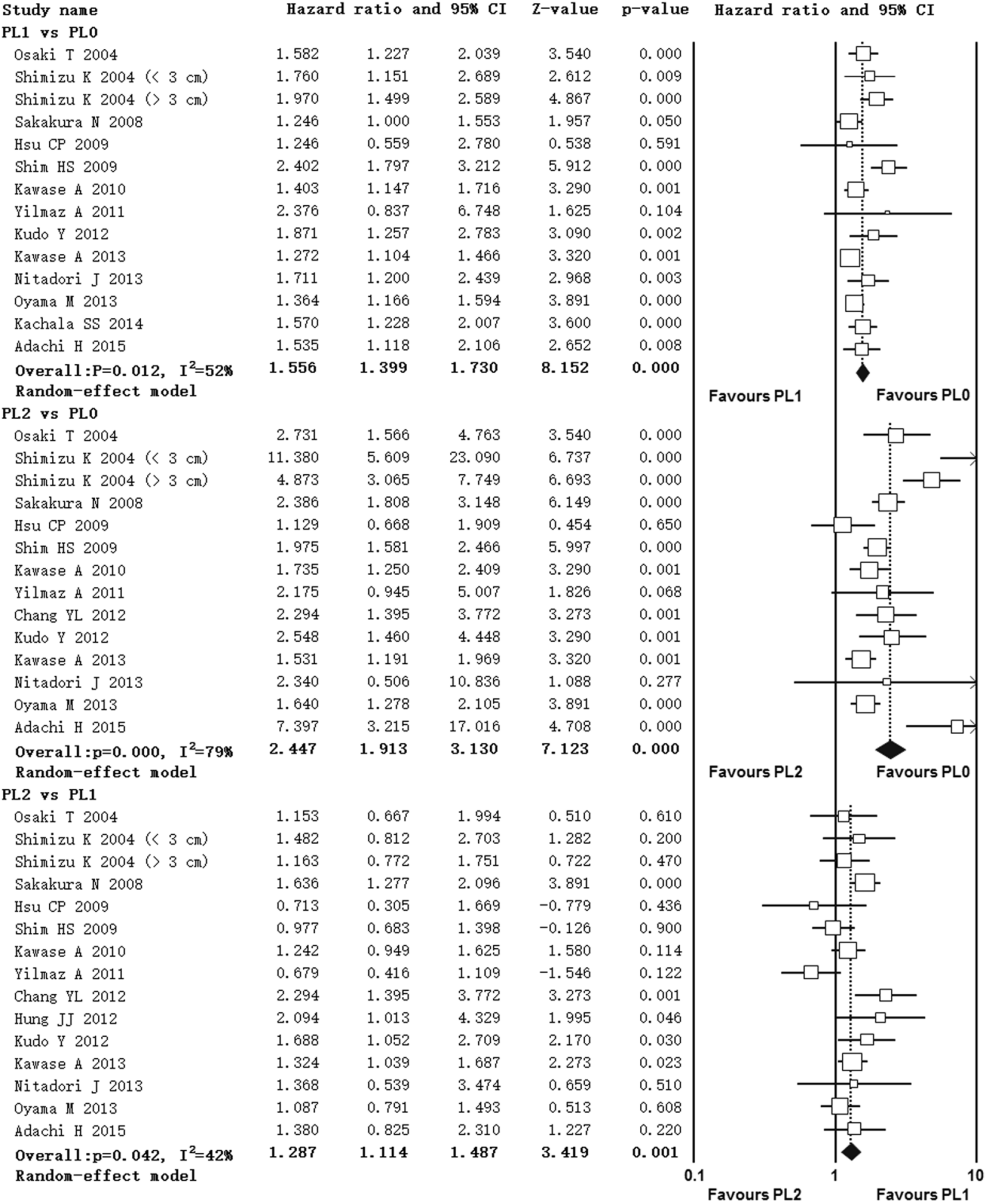
Forest plot showing the impact of extent of VPI on overall survival. ^*^CI: Confidence interval.

Fifteen studies contributed data to the analyses of overall survival^11–24, 32^. Thirteen studies compared overall survival between PL1 and PL0 patients^13–24, 32^, thirteen studies compared PL2 with PL0 patients^11, 13–24^ and fourteen studies compared PL2 with PL1 patients^11–24^. Significant heterogeneity was found among studies in three comparison groups (PL1 vs PL0, I^2^ = 52%, p = 0.012; PL2 vs PL0, I^2^ = 79%, p = 0.000; PL2 vs PL1, I^2^ = 42%, p = 0.042) (Fig. 2). Random-effect model was used. The pooled HR estimate showed that patients with PL1 or PL2 had poorer overall survival compared with PL0 (PL1 vs PL0, HR = 1.555, 95% CI 1.399, 1.730; PL2 vs PL0, HR = 2.447, 95% CI 1.913, 3.130) (Fig. 2). And patients with PL2 had even poorer overall survival than patients with PL1 (HR = 1.287, 95% CI 1.114, 1.487) (Fig. 2).

### Impact of extent of VPI on 5-year survival rate

**Figure 3:**
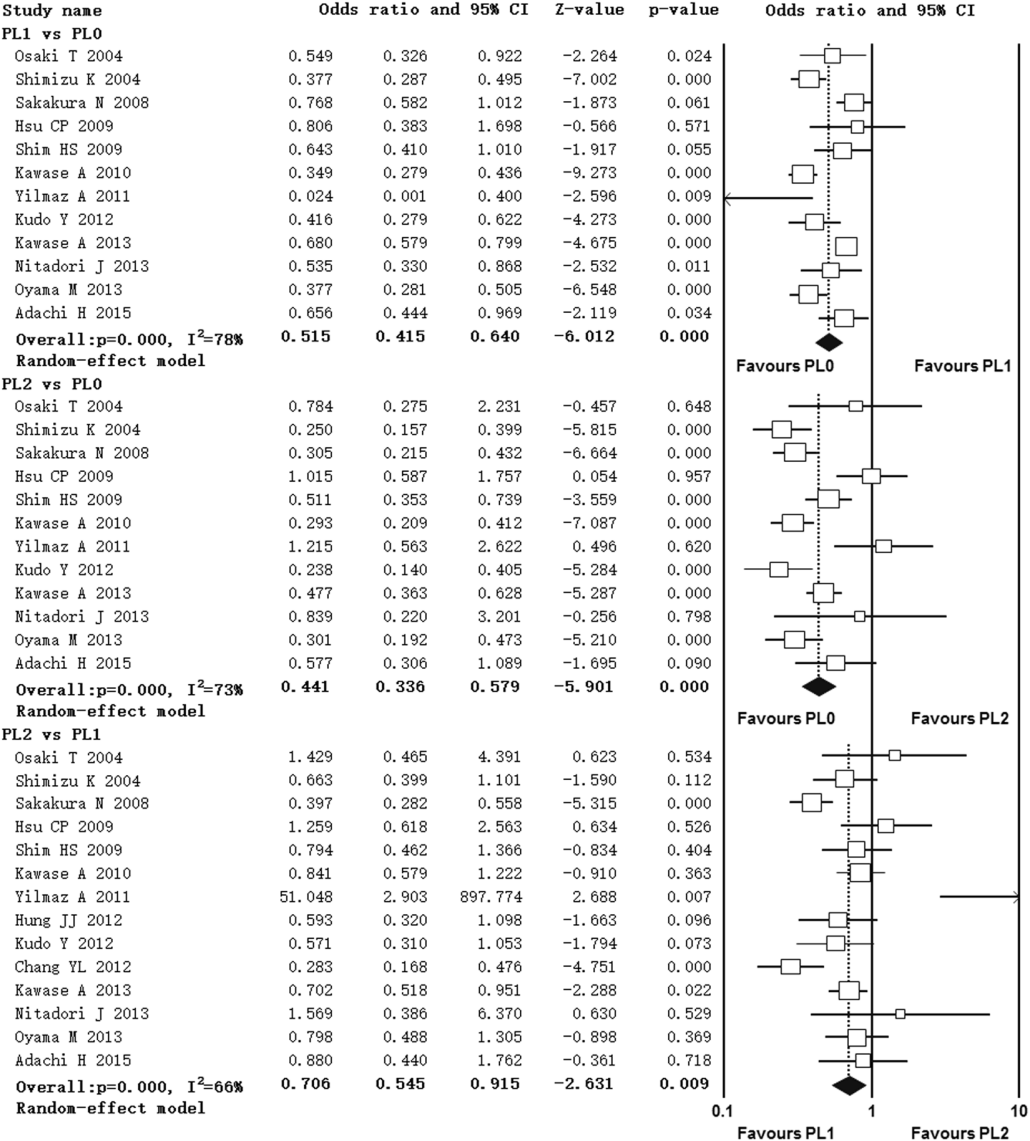
Forest plot showing the impact of extent of VPI on 5-year survival rate. ^*^CI: confidence interval, PTNB: percutaneous transthoracic needle biopsy.

Fourteen studies contributed data to the analyses of 5-year survival rate^11–24, 31^. Twelve studies compared 5-year survival rate between PL1 with PL0 patients^13–24^, twelve studies compared PL2 with PL0 patients^13–24^ and fourteen studies compared PL2 with PL1 patients^11–24, 31^. Significant heterogeneity was found among studies in three comparisons (PL1 vs PL0, I^2^ = 78%, p = 0.000; PL2 vs PL0, I^2^ = 73%, p = 0.000; PL2 vs PL1, I^2^ = 66%, p = 0.000) (Fig. 3). Random-effect model was used. The pooled OR estimate showed that patients with PL1 or PL2 had lower 5-year survival rate than PL0 patients (PL1 vs PL0, OR = 0.515, 95% CI 0.415, 0.640; PL2 vs PL0, OR = 0.441, 95% CI 0.336, 0.579) (Fig. 3). Moreover, PL2 patients had even lower 5-year survival rate than PL1 patients (OR = 0.706, 95% CI 0.545, 0.915) (Fig. 3).

### Impact of extent of VPI on recurrence-free survival

**Figure 4:**
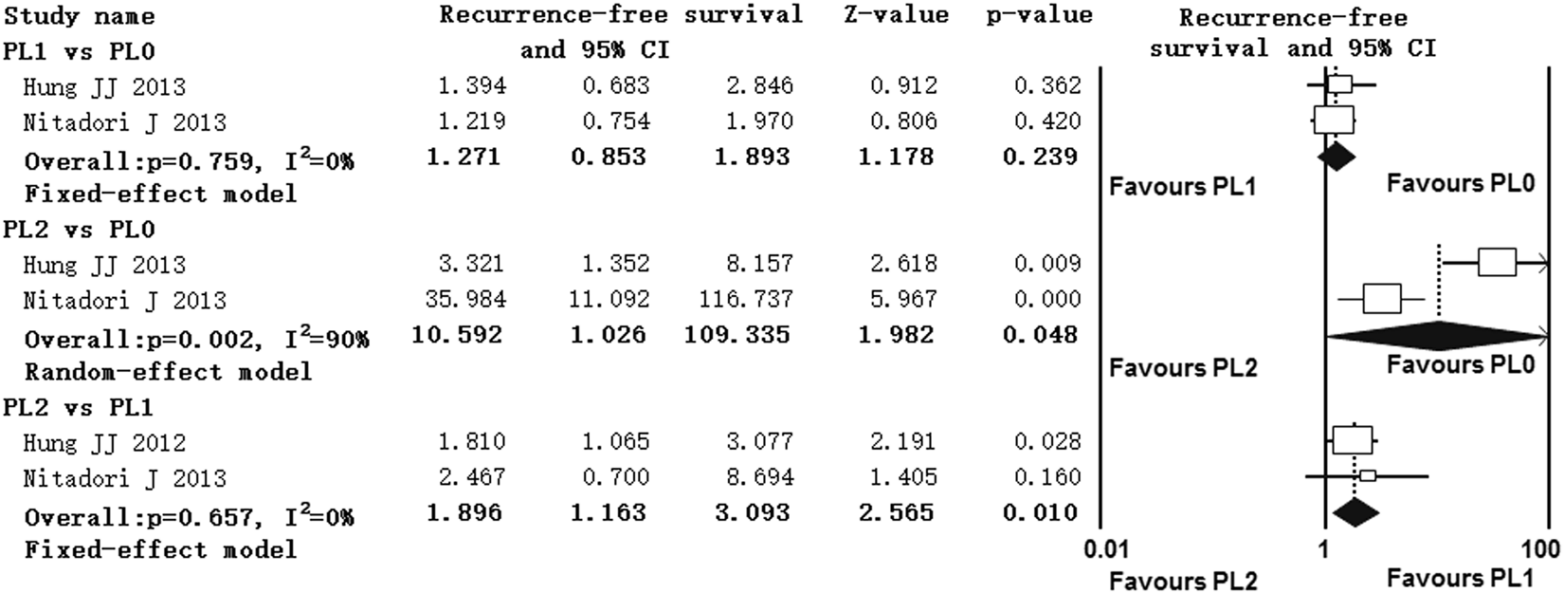
Forest plot showing the impact of extent of VPI on recurrence-free survival. ^*^CI: Confidence interval.

Only three studies contributed data to the analyses of recurrence-free survival^12, 19, 31^. Two studies compared recurrence-free survival between PL1 and PL0 patients^19, 31^, two studies compared PL2 with PL0 patients^19, 31^ and two studies compared PL2 with PL1 patients^12, 19^. Significant heterogeneity was found between studies in PL2 versus PL0 comparison group (I^2^ = 90%, p = 0.002), while significant heterogeneity was not found in PL1 versus PL0 and PL2 versus PL1 comparison groups. The pooled HRs estimate showed that patients with PL1 had similar RFS with PL0 patients (HR = 1.271, 95% CI 0.853, 1.893), patients with PL2 had poorer RFS than PL0 patients (HR = 10.592, 95% CI 1.026, 109.335) and patients with PL2 had poorer RFS than patients with PL1 (HR = 1.896, 95% CI 1.163, 3.093) (Fig. 4).

### Subgroup analyses

When performing the subgroup analyses of overall survival and 5-year survival rate, studies were stratified by confounding factors including staining method, sample size (VPI), follow-up time, tumor stage, and adjuvant therapy. The observed results showed that the deterioration of overall survival and 5-year survival rate in three comparison groups (PL1 vs PL0, PL2 vs PL0, PL2 vs PL1) were identified in most subgroups (Table 2). Negative results were detected only in only 8 of total 60 subgroups which involved no adjuvant therapy, small sample size (<300), short follow-up time (<5 years) or fewer included studies (≤3) (Table 2). For only two included studies of recurrence-free survival the subgroup analyses were not performed on this outcome. All the results of subgroups are shown in Table 2.

**Table 2:** Summarized results of subgroup analyses.

Comparisons		Subgroups	Analysis Model	Comparisons	Heterogeneity		Hazard Ratio			P-value
					P-value	I-square %		95% Confidence Interval		
**Overall Survival**										
PL1 vs PL0	Overall		Random	14	0.012	52	1.556	1.399	1.730	0.000
	Staining method	H&E	Fixed	4	0.483	0	1.317	1.184	1.464	0.000
		H&E and elastic stain	Random	10	0.049	47	1.670	1.469	1.898	0.000
	Sample size (VPI)	<300	Fixed	9	0.497	0	1.811	1.611	2.036	0.000
		>300	Fixed	5	0.596	0	1.344	1.239	1.457	0.000
	Follow-up time	<5 years	Fixed	4	0.879	0	1.636	1.413	1.895	0.000
		≥5 years	Fixed	4	0.733	0	1.332	1.206	1.471	0.000
	Tumor stage	Early	Fixed	3	0.312	14	1.323	1.162	1.507	0.000
		Early and advanced	Random	11	0.022	52	1.602	1.421	1.808	0.000
	Adjuvant therapy	Adjuvant therapy	Fixed	4	0.176	39	1.399	1.253	1.561	0.000
		No adjuvant therapy	Fixed	3	0.617	0	1.680	1.232	2.290	0.001
PL2 vs PL0	Overall		Random	14	0.000	79	2.447	1.913	3.130	0.000
	Staining method	H&E	Random	3	0.014	77	1.671	1.128	2.476	0.011
		H&E and elastic stain	Random	11	0.000	80	2.844	2.082	3.886	0.000
	Sample size (VPI)	<300	Random	9	0.000	82	3.117	1.999	4.859	0.000
		>300	Fixed	5	0.133	43	1.808	1.586	2.061	0.000
	Follow-up time	<5 years	Fixed	3	0.974	0	2.619	1.788	3.834	0.000
		≥5 years	Random	4	0.002	80	1.846	1.207	2.823	0.005
	Tumor stage	Early	Fixed	3	0.491	0	1.461	1.168	1.829	0.001
		Early and advanced	Random	11	0.000	80	2.775	2.104	3.659	0.000
	Adjuvant therapy	Adjuvant therapy	Random	4	0.002	80	2.582	1.501	4.441	0.001
		No adjuvant therapy	Fixed	3	0.342	7	1.444	0.914	2.282	0.115
PL2 vs PL1	Overall		Random	15	0.042	42	1.287	1.114	1.487	0.001
	Staining method	H&E	Random	3	0.130	51	1.374	1.040	1.817	0.026
		H&E and elastic stain	Fixed	12	0.073	40	1.232	1.086	1.397	0.001
	Sample size (VPI)	<300	Fixed	9	0.251	22	1.126	0.950	1.334	0.171
		>300	Fixed	6	0.079	49	1.404	1.238	1.593	0.000
	Follow-up time	<5 years	Fixed	4	0.580	0	1.525	1.126	2.066	0.006
		≥5 years	Fixed	4	0.440	0	1.219	1.022	1.454	0.028
	Tumor stage	Early	Fixed	4	0.312	16	1.327	1.070	1.647	0.010
		Early and advanced	Random	11	0.023	52	1.277	1.073	1.519	0.006
	Adjuvant therapy	Adjuvant therapy	Fixed	4	0.245	28	1.490	1.239	1.792	0.000
		No adjuvant therapy	Fixed	4	0.061	59	0.964	0.685	1.356	0.834
**Five-year survival rate**										
PL1 vs PL0	Overall		Random	12	0.000	78	0.515	0.415	0.640	0.000
	Staining method	H&E	Fixed	3	0.712	0	0.705	0.614	0.808	0.000
		H&E and elastic stain	Random	9	0.020	56	0.447	0.368	0.544	0.000
	Sample size (VPI)	<300	Random	8	0.048	51	0.514	0.403	0.656	0.000
		>300	Random	4	0.000	91	0.513	0.347	0.759	0.001
	Follow-up time	<5 years	Fixed	3	0.629	0	0.483	0.370	0.630	0.000
		≥5 years	Random	4	0.005	77	0.582	0.413	0.821	0.000
	Tumor stage	Early	Fixed	3	0.577	0	0.669	0.576	0.778	0.000
		Early and advanced	Random	9	0.000	76	0.477	0.372	0.612	0.000
	Adjuvant therapy	Adjuvant therapy	Fixed	3	0.084	60	0.638	0.555	0.734	0.000
		No adjuvant therapy	Fixed	3	0.056	65	0.566	0.379	0.846	0.005
PL2 vs PL0	Overall		Fixed	12	0.000	73	0.441	0.336	0.579	0.000
	Staining method	H&E	Random	3	0.001	85	0.508	0.290	0.890	0.018
		H&E and elastic stain	Random	9	0.001	68	0.417	0.300	0.580	0.000
	Sample size (VPI)	<300	Random	8	0.003	67	0.583	0.414	0.822	0.002
		>300	Fixed	4	0.888	0	0.290	0.238	0.354	0.000
	Follow-up time	<5 years	Fixed	3	0.499	0	0.503	0.388	0.652	0.000
		≥5 years	Random	4	0.001	83	0.447	0.235	0.854	0.015
	Tumor stage	Early	Random	3	0.001	86	0.563	0.187	1.693	0.306
		Early and advanced	Random	9	0.002	68	0.414	0.318	0.540	0.000
	Adjuvant therapy	Adjuvant therapy	Random	3	0.047	67	0.405	0.252	0.652	0.000
		No adjuvant therapy	Fixed	3	0.878	0	1.052	0.689	1.607	0.816
PL2 vs PL1	Overall		Random	14	0.000	66	0.706	0.545	0.915	0.009
	Staining method	H&E	Random	3	0.004	82	0.659	0.378	1.147	0.140
		H&E and elastic stain	Random	11	0.004	61	0.732	0.535	1.000	0.050
	Sample size (VPI)	<300	Random	8	0.068	47	0.922	0.631	1.349	0.677
		>300	Random	6	0.002	74	0.568	0.407	0.792	0.001
	Follow-up time	<5 years	Fixed	4	0.317	15	0.700	0.474	1.032	0.072
		≥5 years	Fixed	4	0.508	0	0.786	0.625	0.988	0.039
	Tumor stage	Early	Fixed	4	0.279	22	0.753	0.586	0.967	0.026
		Early and advanced	Random	10	0.000	72	0.675	0.480	0.948	0.023
	Adjuvant therapy	Adjuvant therapy	Random	4	0.017	71	0.558	0.349	0.893	0.015
		No adjuvant therapy	Random	4	0.013	72	1.437	0.529	3.902	0.477

*H&E: hematoxylin-eosin staining; VPI: Visceral pleural invasion.

### Publication bias

**Figure 5:**
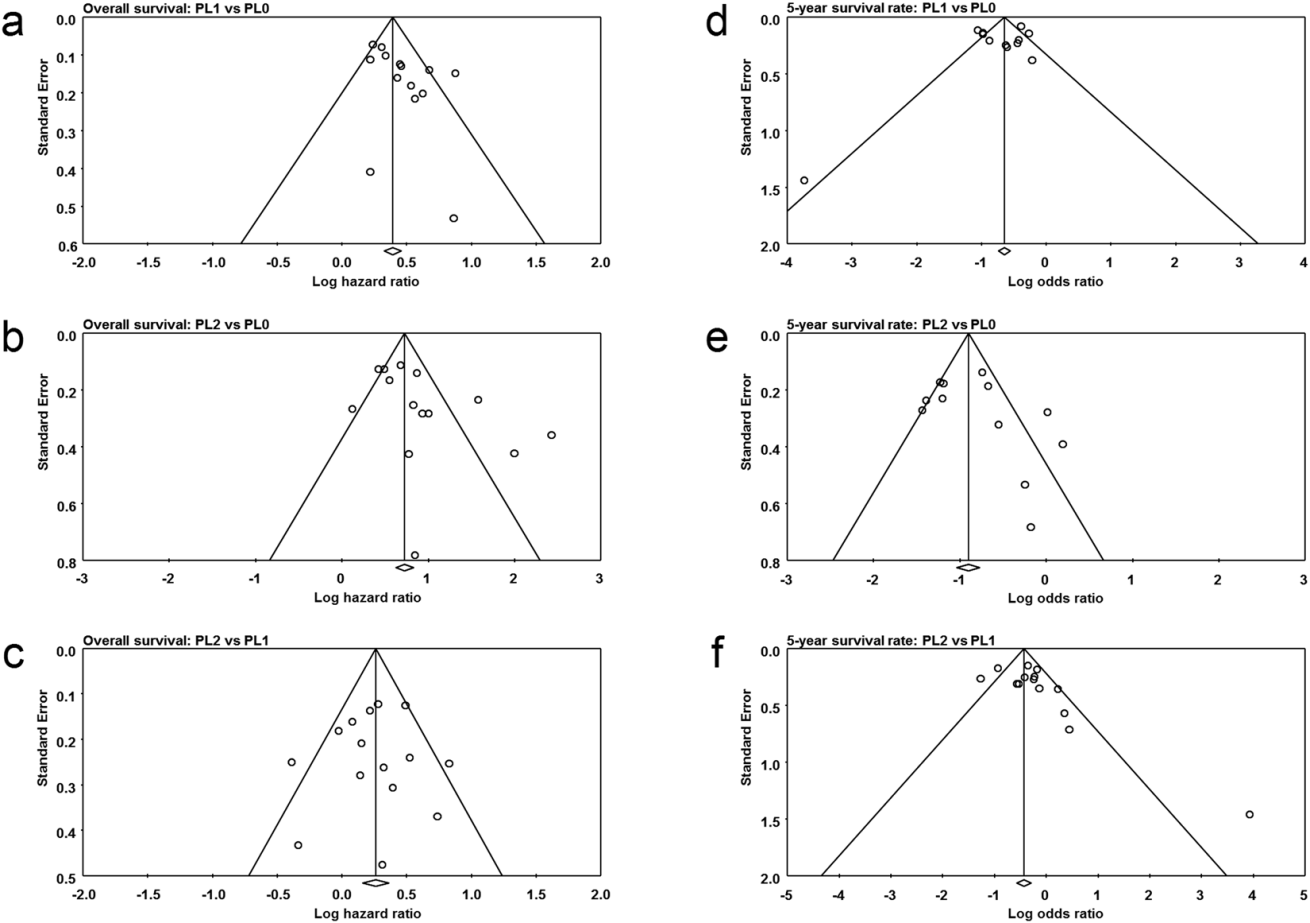
Funnel plots showing the publication bias of overall survival and 5-year survival rate. (**a**) Overall survival: PL1 vs PL0; (**b**) Overall survival: PL2 vs PL0; (3) Overall survival: PL2 vs PL1; (**d**) 5-year survival rate: PL1 vs PL0; (**e**) 5-year survival rate: PL2 vs PL0; (**f**) 5-year survival rate: PL1 vs PL0.

Visual inspection of the funnel plot for OS and 5-year survival rate outcomes did not show the typically asymmetry associated with publication bias. Evidence of publication bias was also not seen with the Bog’s tests of OS and 5-year survival rate (Fig. 5). We were unable to access publication bias of recurrence-free survival owing to the small number of included studies.

### Sensitivity analyses

**Figure 6:**
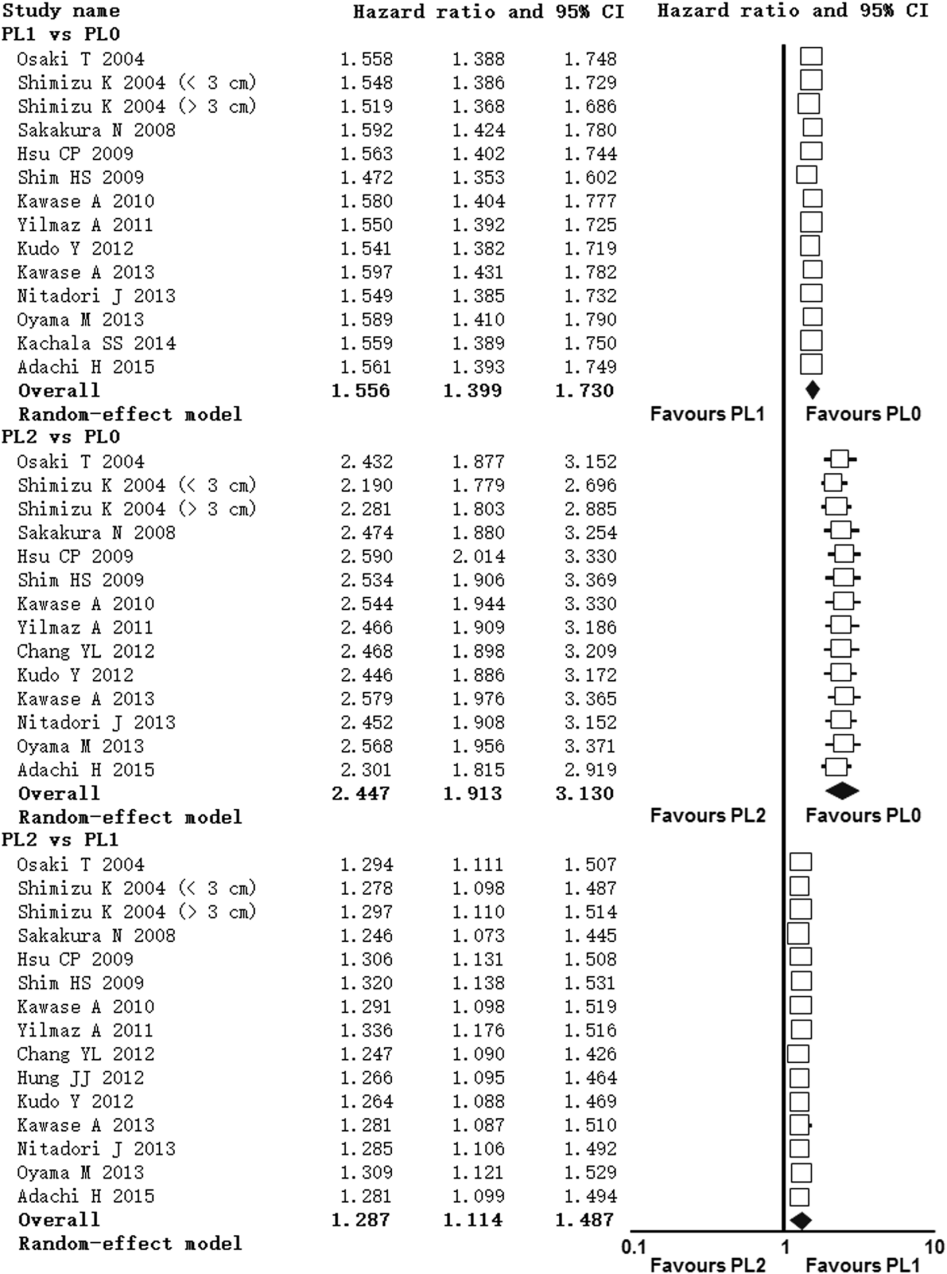
Forest plot showing the sensitivity analyses of overall survival. ^*^CI: Confidence interval.

**Figure 7:**
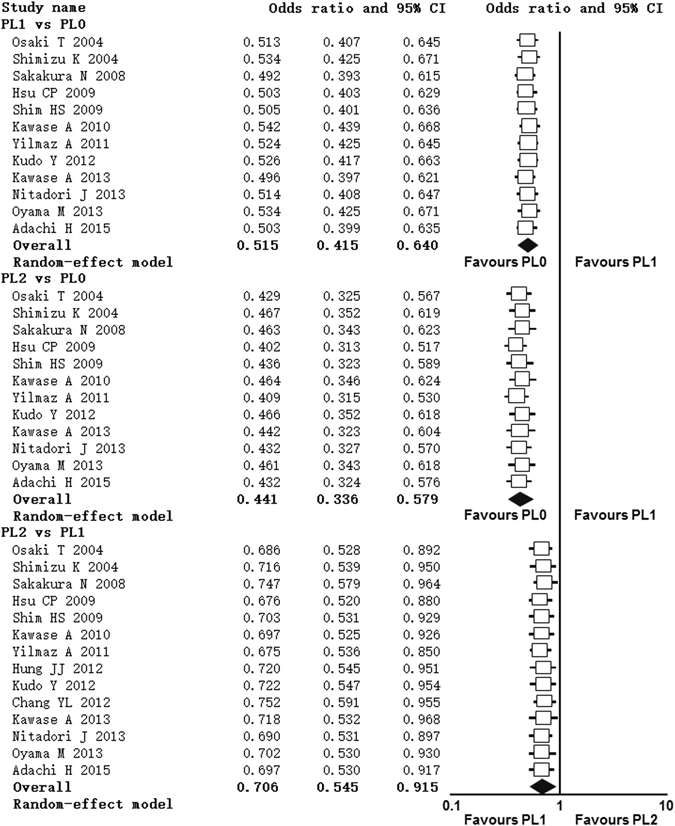
Forest plot showing the sensitivity analyses of 5-year survival rate. ^*^CI: Confidence interval.

The result demonstrated that no individual study had excessive influence on the stability of the pooled effect of each comparison for OS (Fig. 6) and 5-year survival rate (Fig. 7). The result of meta-analysis is robust. For the small number of included studies for RFS, the sensitivity analysis could not be performed.

## Discussion

Visceral pleural invasion, adopted as a T descriptor in the 7th TNM classification of NSCLC, has been reported and constantly studied since 1958^33^. The adverse prognostic significance of VPI in resected NSCLC has been generally reported. The prognostic effect of the extent of VPI, especially PL2 versus PL1, has not been well demonstrated. In this study, we investigated the prognostic role of PL0, PL1 and PL2 on resected NSCLC patients respectively and found patients with PL1 and PL2 had worse OS, 5-year survival rate and RFS than those with PL0. Moreover, patients with PL2 have even worse OS, 5-year survival rate and RFS than those with PL0. Our findings demonstrate that VPI adversely impact the prognosis of resected NSCLC patients differently along with the degree of pleura invasion. These findings are important for further design of studies and for choice of aggressive adjuvant therapeutic strategies.

In the present study, we found significant difference of OS and 5-year survival rate between PL1 and PL0, as well as PL2 and PL0. These findings were consistent with those of our previous study and Jiang L. *et al*.^25, 34^. Our results are also consistent with reported data of the seventh edition and forthcoming eighth edition of the TNM classification of IASLC, published by Rami-Porta *et al*. recently, in which the HR of OS between patients with PL1 and PL0 was 1.44 (95% CI 1.32, 1.58)^4, 35^. Evidence supports that no matter combined as a single category or divided into two categories (PL1 and PL2), VPI was consistently an adverse prognostic factor in resected NSCLC patients. The result of RFS showed a trend but didn’t reach significant survival difference. This reason for this situation may be because only two studies were included. Our results were consistent with the data of forthcoming eighth edition of the TNM classification of IASLC, in which the reported p value of OS for PL2 versus PL1 comparison was 0.012^35^. The poorer prognosis of PL2 than PL1 may result from higher risk of pleural dissemination. Kondo *et al*. reported the pleural lavage cytology was positive in 13 of 96 (14%) and 15 of 41 (37%) of patients in the PL1 and PL2 groups, respectively^36^. Our results may indicate that tumors with PL2 should be upstaged to higher T stage than those with PL1, for example, from T2a to T2b or T2b to T3. Resected NSCLC patients with PL2 may need more aggressive adjuvant treatment.

Because significant heterogeneity was detected we performed subgroup analyses in order to identify confounding factors and source of heterogeneity. For staining method, not all included studies routinely used the elastic stains when detecting VPI. Part of included studies didn’t mention whether elastic stains were used and some of them used elastic stains only when suspicion of VPI. Therefore, there remains some uncertainty regarding the determination of pleura invasion. As noted by Bunker *et al*., the use of an elastic stain is very important for assessing VPI, especially when distinguishing between the PL0 and PL1 status^37^. The subgroup analyses according to staining methods still demonstrated that the survival differences among PL2, PL1 and PL0 remained significant no matter the elastic stain was used or not (Table 2). Besides, the patient numbers of some included studies were small, especially in PL1 and PL2 groups, which might be a reason for negative results of some studies. The subgroup analyses also demonstrated that when comparing PL2 with PL1, the survival difference between patients didn’t reach statistical significance (Table 2). Additionally, some included studies reported follow-up time shorter than 5 years. We performed the subgroup analyses and found that heterogeneity was not significant within subgroups categorized by 5 years. This means follow-up time was an independent confounding factor of survival outcomes. However, no matter in subgroups with follow-up time less than 5 years or in subgroups with follow-up time at least 5 years, the survival differences among PL2, PL1 and PL0 were significant (Table 2).

There are some other limitations of the present study should be mentioned. First, our results are based on low-level evidence from retrospective studies, in most of which some important confounders were not well adjusted, such as tumor size, adjuvant chemotherapy, smoking status or pathologic types. Second, some studies included the patients received incomplete resection that may impact the survival and recurrence, which is also a potential confounder. Third, another potential source of bias is that some HR estimates were derived from reported data or survival curves which involved extrapolation and assumptions. Fourth, of sixteen included studies, only two were from USA and rest were all from Asian countries. The representativeness is limited. In addition, many studies would not include the PL0, PL1 or PL2 factors when performing the multivariate analysis if the result of univariate analysis is not significant. So, pooling these data might have produced bias. Actually, some significant heterogeneity was detected and most of it was unexplainable.

In conclusion, based on available evidence, extent of VPI impacts the prognosis of resected NSCLC and VPI should be categorized as PL1 and PL2 in the terms of clinical practice and trials. Routine elastic tissue staining should be performed as a standard method for assessing pleural involvement in pleura-based NSCLC. However, worldwide, large-scale and prospective studies, in which elastic staining is used as a standard to diagnose VPI status, are warranted.
